# Glyoxalase-I Is a Novel Prognosis Factor Associated with Gastric Cancer Progression

**DOI:** 10.1371/journal.pone.0034352

**Published:** 2012-03-29

**Authors:** Wan-Li Cheng, Ming-Ming Tsai, Chung-Ying Tsai, Ya-Hui Huang, Cheng-Yi Chen, Hsiang-Cheng Chi, Yi-Hsin Tseng, Im-Wai Chao, Wei-Chi Lin, Sheng-Ming Wu, Ying Liang, Chia-Jung Liao, Yang-Hsiang Lin, I-Hsiao Chung, Wei-Jan Chen, Paul Y. Lin, Chia-Siu Wang, Kwang-Huei Lin

**Affiliations:** 1 Department of Biochemistry, Chang Gung University, Taoyuan, Taiwan; 2 Department of Nursing, Chang-Gung University of Science and Technology, Taoyuan, Taiwan; 3 Medical Research Center, Chang Gung Memorial Hospital, Taoyuan, Taiwan; 4 Proteomics Core Laboratory, Chang Gung University, Taoyuan, Taiwan; 5 First Cardiovascular Division, Chang Gung Memorial Hospital, Taoyuan, Taiwan; 6 Department of Pathology, Chang Gung Memorial Hospital, Chiayi, Taiwan; 7 Department of General Surgery, Chang Gung Memorial Hospital, Chiayi, Taiwan; Vanderbilt University Medical Center, United States of America

## Abstract

Glyoxalase I (GLO1), a methylglyoxal detoxification enzyme, is implicated in the progression of human malignancies. The role of GLO1 in gastric cancer development or progression is currently unclear. The expression of GLO1 was determined in primary gastric cancer specimens using quantitative polymerase chain reaction, immunohistochemistry (IHC), and western blotting analyses. GLO1 expression was higher in gastric cancer tissues, compared with that in adjacent noncancerous tissues. Elevated expression of GLO1 was significantly associated with gastric wall invasion, lymph node metastasis, and pathological stage, suggesting a novel role of GLO1 in gastric cancer development and progression. The 5-year survival rate of the lower GLO1 expression groups was significantly greater than that of the higher expression groups (log rank *P* = 0.0373) in IHC experiments. Over-expression of GLO1 in gastric cancer cell lines increases cell proliferation, migration and invasiveness. Conversely, down-regulation of GLO1 with shRNA led to a marked reduction in the migration and invasion abilities. Our data strongly suggest that high expression of GLO1 in gastric cancer enhances the metastasis ability of tumor cells *in vitro* and *in vivo*, and support its efficacy as a potential marker for the detection and prognosis of gastric cancer.

## Introduction

Gastric cancer is the fourth most common form of cancer and the second highest cause of cancer-related mortality worldwide [1]. The malignancy is the sixth leading cause of cancer-related deaths in Taiwan [2]. Proper screening can facilitate the detection of gastric cancer before symptom development at a curable stage [3]. Determination of the expression profiles of key molecules in the several pathways involved in gastric cancer progression may aid in diagnosis, prognosis and prediction of tumor progression.

Tumor invasion and metastasis are vital steps in determining the aggressive phenotype of human cancers, and constitute the principal causes of cancer-related death [4]. High expression of migration-related factors, such as cyclooxygenase 2 (COX-2) [5], Vascular endothelial growth factor (VEGF) [6], CXC chemokine ligand (CXCL)-8 [7], chemokine (C-X-C motif) receptor (CXCR)-2, and CXCL-1 [8], are associated with gastric cancer progression. Several potential oncogenic pathways (proliferation/stem cell, NF-κB, and Wnt/β-catenin) are deregulated in the majority of gastric cancers [9]. Thus, further elucidation of the exact molecular events leading to gastric cancer progression and identification of valuable diagnostic or prognostic markers and novel therapeutic strategies would be of significant clinical value.

Glyoxalase I (also termed GLO1) is an essential component in pathways leading to the detoxification of Methylglyoxal (MG), one of the side products of glycolysis [10], [11], [12]. GLO1 expression is increased in several human cancers of the colon, breast, prostate, and melanoma [13], [14], [15], [16], [17]. Recent studies have reported that over-expression of GLO1 is associated with cancer progression and drug resistance [17]. From our previous data, GLO1 upregulation was observed in gastric cancer specimens using cDNA microarray [18]. However, the specific role of GLO1 during gastric tumorigenesis and its clinical significance remain to be established.

Our experiments clearly show that GLO1 is frequently over-expressed in gastric cancer and associated with cancer metastasis. Notably, expression of GLO1 is significantly higher in advanced stages of gastric cancer. Furthermore, alterations the expression of GLO1 in gastric cancer cell lines affects cell migration and invasion abilities.

## Materials and Methods

### Ethics Statement

The study protocol was approved by the Medical Ethics and Human Clinical Trial Committee of the Chang Gung Memorial Hospital (IRB NO. 95-0472B). Written informed consent was obtained from all patients.

### Subjects

The 114 patients (64 males and 50 females; median age, 66 years; range 28–86 years) diagnosed with gastric cancer at the Chang Gung Memorial Hospital from 2000 to 2005 were enrolled in this study. All patients received surgery for primary gastric cancer without prior chemotherapy or radiotherapy. Each patient was subjected to gastric resection (35 patients underwent total gastrectomy and 79 partial gastrectomy).

### Clinicopathological studies

Resected specimens were examined pathologically using the criteria of the Japanese General Rules for Gastric Cancer Study [19] and the American Joint Committee on Cancer (AJCC) (pTNM) classification system [20]. Clinicopathological parameters included patient age and gender, tumor location and size, gross (Borrmann's) tumor type, wall invasion, resection margin, histological type, lymph node metastasis, vascular invasion, lymphatic invasion, and perineural invasion. After discharge, all patients had periodic follow-up visits at the outpatient department of Chang Gung Memorial Hospital until death or the beginning of preparation of this article.

### Real-time quantitative reverse transcription polymerase chain reaction (qRT–PCR)

qRT–PCR was performed as described in a previous report [21]. The following primers were used: human *GLO1* qRT–PCR (forward primer, 5′–TGAGGATAAAAATGACATCCCTA- AAGA–3′, and reverse primer, 5′–TGTGTCAGCTCAAGTGTAGCTTTC–3′), human 18S rRNA qRT–PCR (forward primer, 5′–CGAGCCGCCTGGATACC–3′, and reverse primer, 5′–CCTCAGTT CCGAAAACCAACAA–3′).

### Production of anti-GLO1 antibody

The cDNA encoding full-length *GLO1* was cloned into pGEX-4T1. Lysates from *E. coli* BL21 strain were purified with glutathione-agarose beads (Sigma-Aldrich, St. Louis, MO). Soluble proteins were purified using chromatography with glutathione-agarose beads, according to the manufacturer's instructions, emulsified with adjuvant, and used to immunize rabbits. Polyclonal antibodies were produced and affinity-purified, as described previously [22]. The specificity of in-house GLO1 was validated using western blot analysis (Figure S1).

### Immunoblot analysis

Whole cell lysates, nuclear extracts, and conditional media were prepared from human tissue or stable GLO1 knockdown cell lines. Western blotting was performed using monoclonal antibodies against human HIF-1α (Abcam, San Francisco, CA), p65 (Epitomic, Burlingame, CA), or p50 (Millipore, Billerica, MA) or polyclonal antibodies against human GLO1 (in-house, dilution, 1∶500), CXCL1 (PeproTech. Inc., Rocky Hill, NJ), CXCL8 (R&D Systems Inc., Minneapolis, MN), VEGF (Santa Cruz Biotechnology, Santa Cruz, CA).

### Immunohistochemistry (IHC)

Formalin-fixed and paraffin-embedded tissues were examined with IHC using the polyclonal antibody against human GLO1 produced in-house (dilution, 1∶3000) and the avidin–biotin complex (ABC) method, as described previously [23], [24]. Comparisons were performed between the intensity of staining of carcinoma cells and benign superficial epithelium, which were placed on the same slide. For semi-quantitative analysis of GLO-1 immunoreactivity, a Histoscore (H)-scoring system was used [25]. Briefly, the negative group consisted of cancer cells with no detectable (−) or only trace staining for GLO-1 (+1). The positive group consisted of cancer cells with moderate (+2) or high levels (+3) of GLO-1 immunoreactivity. The H-scoring was calculated and averaged by two independent pathologists, blinded to the initial score for each patient. The results were scored by multiplying the percentage of positive cells (P) by the intensity (I), according to the formula: H = P×I. For example, a section in which 10% of the tissue had a staining score of +1, 60% a score of +2, and 30% a score of +3, H = (10×1)+(60×2)+(30×3) = 220.

### Establishment of GLO1 over-expression in SC-M1 cell line

The SC-M1 cell line expressing lower level of GLO1 was used. The transfection of *GLO1* cDNA was performed with Lipofectamine Reagent (Life Technologies, Grand Island, NY). After incubation for 24 h, the cells were transferred to medium containing G418 for selection, and were then used in proliferation, migration, and invasion assays.

### Establishment of GLO1 knockdown in TSGH and AGS cell lines

Two human gastric cancer cell lines, AGS, and TSGH, were employed. The short hairpin RNA (shRNA) sequences targeting *GLO1* (TRCN0000118630 and TRCN0000118631) were purchased from the National RNA Interference Core Facility (Institute of Molecular Biology, Academia Sinica, Taiwan). The specific repression of GLO1 was confirmed using western blot analysis.

### Cell proliferation assay

Cells (1×10^4^) were grown on a 6 cm plate at 37°C under 5% CO_2_. At each time point, the growth rate of the cells was determined by cell counting. The results are given as the fold change relative to each control value.

### *In vitro* assay of migration and invasive activity

The effect of GLO1 depletion or over-expression on the migration and invasive activity of gastric cancer cell lines was assessed using a rapid *in vitro* assay (Transwell technique), as described previously [26].

### RNA preparation and microarray analysis

The GLO1-silenced clone TSGH (KG2) and control cell clone (C1) were rinsed briefly with ice-cold PBS and lysed in TRIzol reagent (Invitrogen) for RNA extraction. Gene expression profiles between KG2 and C1 cells were analyzed with the human U133A GeneChip (Affymetrix, Santa Clara, CA) according to the manufacturer's protocol [27].

### Statistical analysis

The GLO1 expressions of each subgroup of clinicopatholgoical parameters in Table 1 are expressed as mean ± standard deviation (SD) of the IHC score of the patients in this subgroup. The Kolmogorov-Smirnov test is a nonparametric test to compare samples with a reference probability distribution. Where appropriate, the Mann-Whitney U or Fisher's exact test was applied for comparisons between the two groups, while Kruskal-Wallis or Pearson's chi-square test was used to compare more than two groups. The relationship between data obtained from the two different examinations was analyzed with the Spearman's correlation test. Patients were monitored until the time of manuscript preparation or death. Cancer-specific survival outcome was determined by applying the Kaplan-Meier method for all patients, except those who died from surgical complications. The log-rank test was employed to compare the prognostic significance of individual variables on survival. The Cox proportional hazards model was employed in multivariate analysis to identify the independent predictors of survival. *P* values <0.05 were considered significant.

**Table 1:** Clinicopathological Correlations of GLO1 expression and 5-year Survival Rate in 114 Gastric Cancer Patients.

Parameters	No.	IHC GLO1^1^	*P* value^2^	5-yr S.R.^3^	logrank *P* 4
**Age (yrs)**					
<65	55	129.6±57.1	0.096	45.5	0.4133
≥65	59	149.3±66.8		53.2	
**Gender**					
Male	64	147.3±59.9	0.178	47.4	0.4958
Female	50	130.2±65.7		51.5	
**Location**					
Upper third	26	136.9±65.1	0.679	53.9	0.4288
Middle third	26	129.6±61.9		48.7	
Lower third	59	143.7±60.6		49.2	
Whole	3	176.7±107.9		0	
**Gross type**					
Localized	43	127.2±60.7	0.097	75.8	<0.0001
Infiltrative	71	147.5±63.3		33.1	
**size**					
<5 cm	58	130.2±55.6	0.158	71.0	<0.0001
≥5 cm	56	149.8±68.6		25.1	
**Histological type**					
Intestinal	36	141.7±59.3	0.555	73.9	0.0002
Diffuse	78	139.0±64.7		37.2	
**Depth of invasion**					
T1,2	42	113.6±58.2	0.001	82.8	<0.0001
T3,4	72	115.1±60.6		28.8	
**Lymph node metastasis**					
No	31	108.1±63.0	<0.001	92.2	<0.0001
Yes	83	151.7±58.8		33.6	
**Distant metastasis**					
No	85	134.2±58.7	0.277	63.5	<0.0001
Yes	29	156.2±72.2		0.0	
**Pathological stage**					
Stages 1,2	39	103.9±52.2	<0.001	89.9	<0.0001
Stages 3,4	75	158.5±59.9		27.0	
**Liver metastasis**					
No	112	139.0±62.8	0.352	50.2	0.0267
Yes	2	185.0±63.6		0.0	
**Peritoneal seeding**					
No	93	137.7±60.1	0.758	59.0	<0.0001
Yes	21	149.1±74.8		0.0	
**Vascular invasion**					
No	89	133.8±57.9	0.144	57.6	0.0001
Yes	25	161.2±75.4		8.2	
**Lymphatic invasion**					
No	47	123.0±67.9	0.016	77.2	<0.0001
Yes	67	151.6±56.6		28.7	
**Perineural invasion**					
No	71	133.0±62.9	0.164	62.0	0.0012
Yes	43	151.2±61.7		25.4	
**GLO1 (IHC score)**					
<90	24	55.8±20.2		69.6	0.0373
≥90	90	162.2±49.9		43.3	

1 IHC scores of GLO1; in mean±standard deviation.

2 Mann-Whitney U test (for 2 groups) or Kruskal Wallis test (for >2groups).

3 5-year survival rate.

4 Log rank test.

## Results

### GLO1 mRNA and protein levels are upregulated in gastric cancer patients

Using cDNA microarray, we have identified several upregulated genes from gastric tissues, compared to adjacent nontumorous tissues [18]. Among these genes, we focused on *GLO1* as a molecular target for gastric cancer. Expression of GLO1 was measured in cancer tissues, and compared with that in matched nontumorous gastric mucosa, using qRT–PCR (n = 89) (Table 2) or IHC (n = 114) (Table 1). Data from qRT-PCR experiments revealed GLO1 overexpression (≥1.5-fold) in 51 (57.3%) of gastric cancer tissues, compared with noncancerous tissues. The mean GLO1 expression in tumor tissues was 2.87-fold that in noncancerous tissues. Our results confirmed a significant increase in GLO1 expression in tumor tissues (*P* = 0.005, one-sample Kolmogorov-Smirnov test).

**Table 2:** Clinicopathological Correlations of GLO1 expression (Q-RT-PCR) in 89 Gastric Cancer Patients.

Parameters	No.	GLO1^1^	*P*value^2^	Parameters	No.	GLO1^1^	*P*value^2^
**Age (yrs)**				**Lymph node** **metastasis**			
<65	46	2.6±3.2	0.386	No	29	1.4±0.9	0.001
≥65	43	3.1±3.0		Yes	60	3.5±3.6	
**Gender**				**Distant metastasis**			
Male	51	2.8±3.3	0.816	No	66	2.5±2.3	0.616
Female	38	2.8±2.9		Yes	23	3.9±4.7	
**Location**				**Pathological stage**			
Upper third	23	2.7±2.8	0.656	Stages 1,2	33	1.4±0.9	0.001
Middle third	19	2.3±2.7		Stages 3,4	56	3.7±3.6	
Lower third	42	3.2±3.6		**Liver metastasis**			
Whole	5	2.2±1.7		No	87	2.9±3.2	0.923
**Gross type**				Yes	2	2.0±1.4	
Localized	37	1.9±1.7	0.043	**Peritoneal seeding**			
Infiltrative	52	3.5±3.7		No	71	2.4±2.1	0.176
**Size**				Yes	18	4.8±5.2	
<5 cm	48	1.9±1.7	0.014	**Vascular invasion**			
≥5 cm	41	3.9±4.0		No	70	2.8±3.2	0.548
**Histological type**				Yes	19	3.0±3.0	
Intestinal	31	2.6±2.2	0.624	**Lymphatic invasion**			
Diffuse	58	3.0±3.5		No	38	1.6±1.3	0.001
**Depth of invasion**				Yes	51	3.8±3.7	
T1,2	35	1.7±1.3	0.015	**Perineural invasion**			
T3,4	54	3.6±3.7		No	58	2.2±2.2	0.024
				Yes	31	4.0±4.1	

1 Folds: measured by real time Q-RT-PCR in tumor tissues compared to adjacent non-tumor tissues; in mean±standard deviation.

2 Mann-Whitney U test (for 2 groups) or Kruskal Wallis test (for >2 groups).

**Figure 1:**
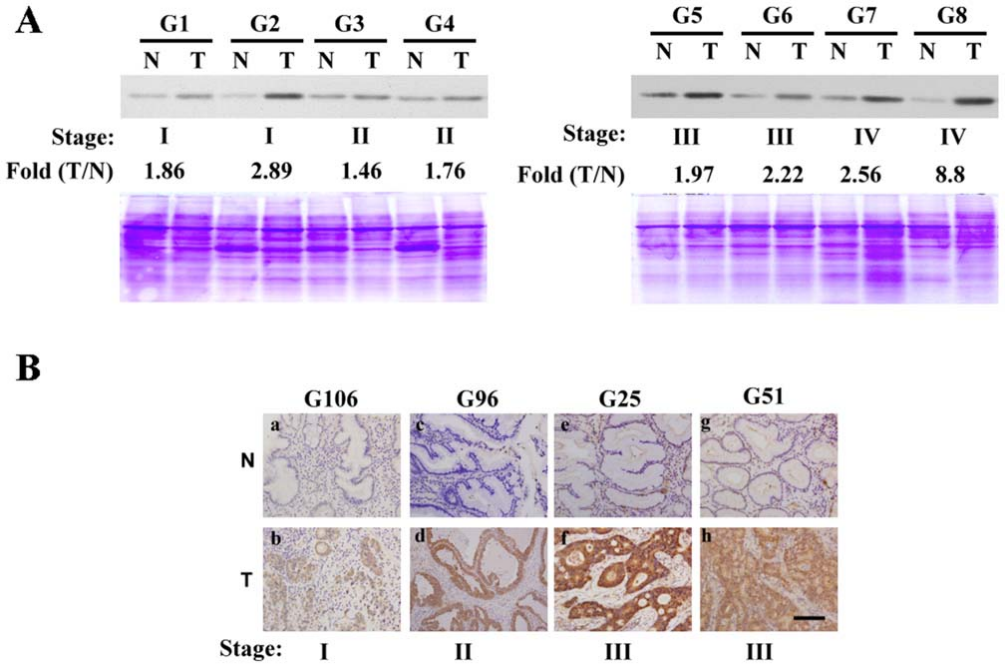
GLO1 expression is elevated in human gastric carcinoma. Overexpression of GLO1 protein in gastric carcinoma. (A) Western blot demonstrating the presence of GLO1 protein in cancer tissues. GLO1 proteins were overexpressed in most tumor tissues (T), compared with matched noncancerous adjacent mucosa (N). All cancer tissues from gastric cancer specimens (G1 to G8) displayed upregulation of GLO1, compared with matched noncancerous adjacent mucosa. An equivalent amount (30 µg) of protein was loaded for each specimen and subjected to 12% SDS-PAGE, followed by staining with Coomassie blue as a loading control. Rabbit polyclonal antibody against human GLO1 produced in-house was used. (B) Panels a, c, e, and g depict noncancerous mucosa, while panels b, d, f, and h depict gastric cancer tissues. Positive staining for GLO1 is indicated as a dark-brown color. GLO1 expression was observed predominantly in gastric cancer cells and rarely in stromal cells. Scale bar represents 100 µm.

Expression of GLO1 protein in paired specimens was further analyzed using western blotting. Figure 1A presents GLO1 expression in eight representative patients. Equal amounts of total proteins stained with Coomassie blue after SDS-PAGE were used as the loading control. All cancer tissues from gastric cancer samples (G1 to G8) displayed upregulated GLO1 expression, compared with matched noncancerous adjacent mucosa (Fig. 1A).

### Immunostaining demonstrates GLO1 protein over-expression in gastric cancerous tissues

To further establish whether GLO1 upregulation is correlated with clinical progression of gastric cancer, IHC was performed on paraffin-fixed gastric cancer tissues and matched noncancerous mucosa of 114 patients. Four pairs of representative cases (a/b, c/d, e/f, and g/h) are shown in Figure 1B. IHC data for noncancerous mucosa counterparts (a, c, e, and g) and cancer tissues (b, d, f, and h) were compared in pairs. Dark-brown immunostaining was mostly prevalent in cancer cells whereas levels of staining were lower in stromal cells or fibroblasts of gastric cancer tissues. Strong staining for GLO1 was frequently observed in advanced gastric tumor cells, in contrast to weak or no staining in normal gastric epithelial cells (Fig. 1B, upper panel). Staining was more intense at the advanced gastric cancer stages [stage III in Fig. 1B (f, h)], compared with stages I [Fig. 1B (b)] and II [Fig. 1B (d)]. Among the 114 patients analyzed, the mean IHC score in tumor tissues was 139.8±62.8, which was significantly greater than that (36.7±40.4) in the matching adjacent mucosa (n = 87) (*P*<0.001, Wilcoxon signed-rank test). Furthermore, paired comparison of immunoreactivity for GLO1 (n = 87) revealed that the IHC scores of cancerous tissues were higher than those of the nontumorous counterparts in 77 (88.5%) patients, equal in three patients (3.4%), and lower in seven patients (8.0%).

### GLO1 expression and clinical correlations

**Figure 2:**
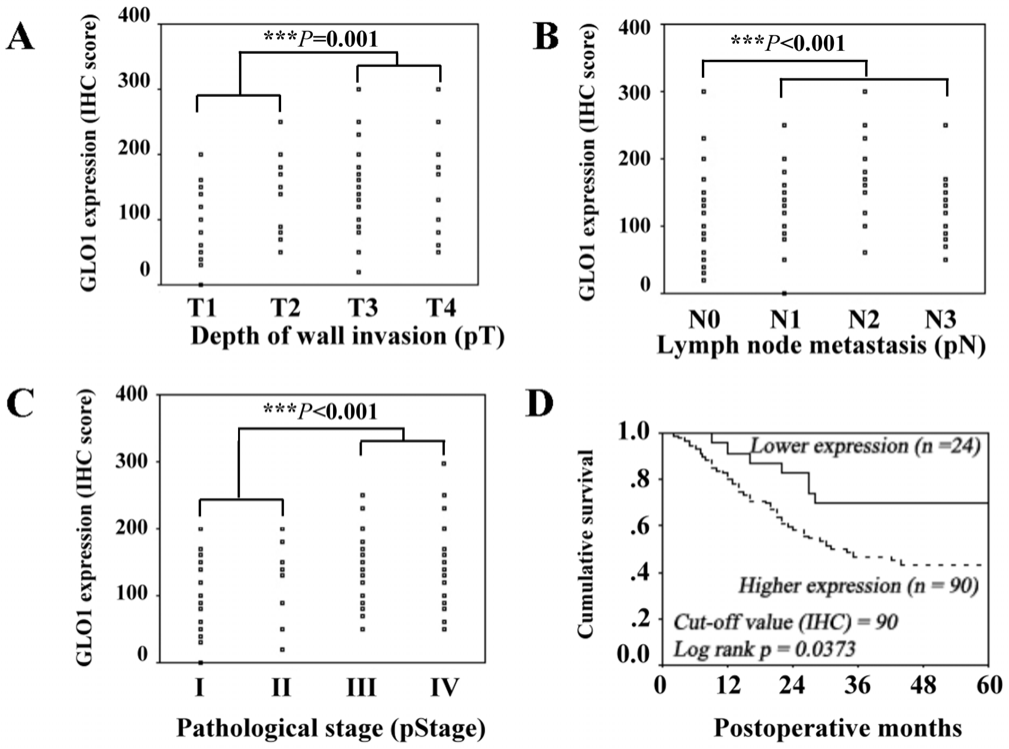
Scatter plots of IHC scores of GLO1 and various clinicopathological features. (A) Scatter plot according to depth of wall invasion (*P* = 0.001, T1/T2 vs. T3/T4). (B) Scatter plot according to lymph node metastasis (*P*<0.001, N0 versus N1–3). (C) Scatter plot according to pathological stage (*P*<0.001, stages I/II versus stages III/IV). (D) Kaplan-Meir survival curves of two groups of gastric cancer patients defined by a GLO1 expression level cutoff value of 90, established on the basis of IHC scoring. The 5-year survival rate of the lower expression groups (n = 24) was significantly better than that of the higher expression groups (n = 90; 69.6% vs. 43.3%; log rank *P* = 0.0373).

GLO1 expression in tumor tissue was not significantly associated with age, tumor location or histological type (Tables 1 and 2). Higher levels of GLO1 were evident in the T3/T4 groups where the serosal surface of the gastric wall was invaded by cancer, compared to that in T1/T2 groups where no invasion was evident (*P* = 0.015 for qRT- PCR and *P* = 0.001 for IHC; Fig. 2A; Tables 1 and 2). Expression of GLO1 was significantly increased, with metastasis to the lymph nodes (*P* = 0.001 for qRT-PCR and *P*<0.001 for IHC; Fig. 2B; Tables 1 and 2). Higher expression was evident in patients with lymphatic invasion (*P* = 0.001 and *P* = 0.016 for qRT-PCR and IHC respectively; Tables 1 and 2) and perineural invasion (*P* = 0.024 for qRT-PCR, Table 2). Increased GLO1 expression was not associated with vascular invasion or distant metastasis, including peritoneal seeding or liver metastasis, in both qRT-PCR and IHC experiments. Expression of GLO1 was significantly higher in patients with more advanced pathologic stages (III/IV) of gastric cancer, compared to those in the earlier pathologic stages (I/II) (*P* = 0.001 and *P*<0.001 for qRT-PCR and IHC, respectively) (Fig. 2C; Tables 1 and 2).

### Survival outcomes

The mean duration of the follow-up period for 52 survivors was 70.4 months (range, 28–119 months). Four patients died of postoperative complications and six of other causes. Fifty-two patients died owing to gastric cancer progression. The overall cumulative five-year survival rate of the 114 patients was 49.3% after gastrectomy. To determine the influence of GLO1 expression on survival outcome, the patient was divided into two groups, higher and lower expressions, according to the cutoff value which would demonstrate a significant difference (log rank *P*<0.05) in survival rates between 2 groups. The median ( = 140), upper quartile ( = 180 or 75^th^ percentile), and lower quartile ( = 90 or 25^th^ percentile) of IHC scores of our patient were initially tested to determine the cutoff values. Among them, only the lower quartile could show a significant difference in survival outcome. Fig. 2D illustrates the cumulative survival curves of patients in the lower and higher expression GLO1 groups, divided according to a cutoff IHC score of 90. The 5-year survival rate of the lower GLO1 expression groups was significantly greater than that of the higher expression groups (69.6% vs. 43.3%; log rank *P* = 0.0373) in IHC experiments. Univariate analysis disclosed a number of significant prognostic factors, including status of lymph node metastasis, distant metastasis, peritoneal seeding, vascular invasion, lymphatic invasion, depth of invasion, pathological stage, liver metastasis and perineural invasion, in addition to GLO1 expression. Other significant parameters were histological type, tumor size, and gross type (Table 1). Further, in multivariate analysis, the independent prognostic factors influencing patient survival included lymph node metastasis (relative risk = 5.954, 95% CI = 1.183–29.977, *P* = 0.031) and distant metastasis (relative risk = 2.464, 95% CI = 1.030–5.896, *P* = 0.043).

#### Over-expression of GLO1 in SC-M1 enhances cell proliferation, migration and invasion activities

**Figure 3:**
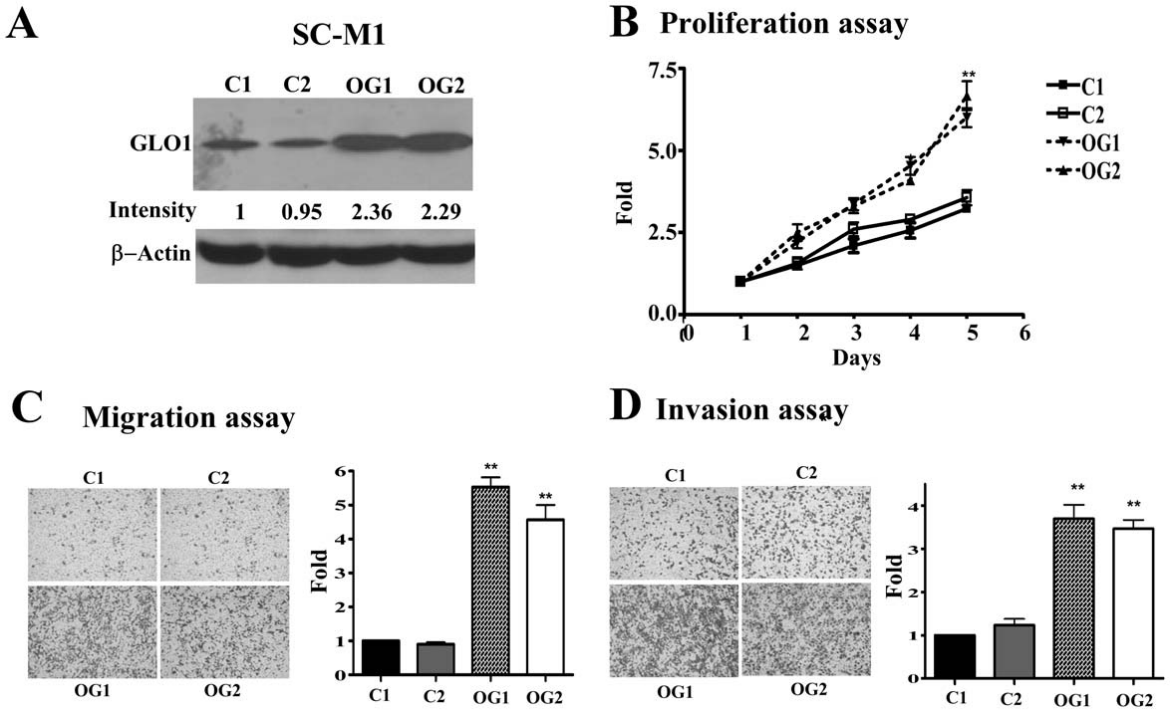
Effects of GLO1 over-expression in SC-M1 cells. Two SC-M1-GLO1 over-expression clones (OG1 and OG2), and two control cell lines (C1, C2) were established. (A) Expression of GLO1 was determined using western blot analysis. β-actin was used as an internal control. (B) Cell proliferation, (C) Migration, and (D) Invasion abilities were assayed as described in “Materials and Methods”. Data were presented as folds from at least three independent experiments performed in duplicated. The fold changes (B–D), and differences examined using Mann-Whitney U method to compare values with vector control. ** *P*<0.05.

To determine the effects of over-expression of *GLO1* in SC-M1 cells, cell proliferation, migration, and invasion activities were assayed. After two weeks of transfection, stable expression of GLO1 protein was established. Figure 3A shows 2.36 fold and 2.29 fold higher GLO1 expression, respectively. Cell proliferation was determined by cell counting and indicated as a fold of the control for up to five days. *GLO1*-overexpressing cells exhibited significantly (*P*<0.01) higher proliferation rates (1.86- or 2.06-fold) than those transfected with control vector on day 5 (Fig. 3B). Moreover, *GLO1*-overexpressing cells displayed significantly (*P*<0.01) higher migration rates (5.53- or 4.57-fold) and invasive abilities (3.7- or 3.47-fold) than their control counterparts (Figs. 3C and D). Images of cell density were shown for two control and two over-expressing cell lines (left panels in Figs. 3C and D).

### Down-expression of GLO1 in TSGH or AGS cells reduces cell migration and invasion activities

**Figure 4:**
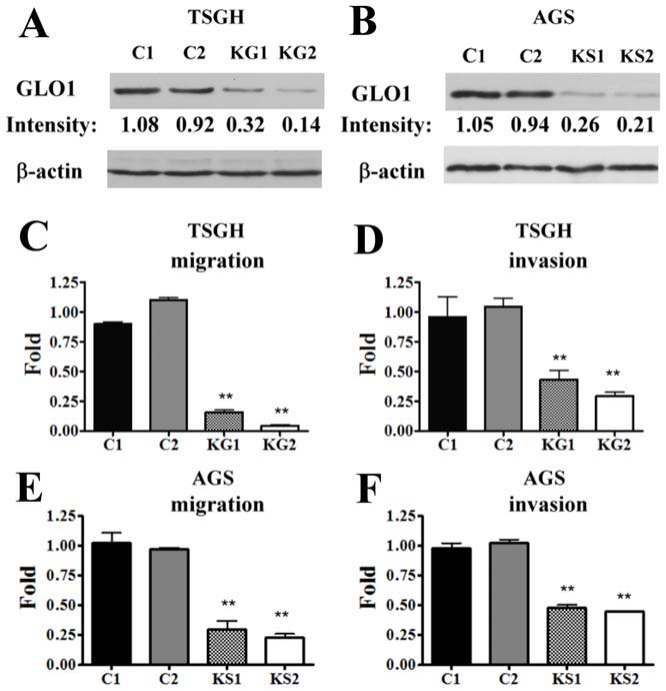
Knockdown of GLO1 expression suppresses TSGH or AGS cell migration and invasion. Two TSGH-GLO1-silenced clones (KG1 and KG2), two AGS-GLO1-silenced clones (KS1 and KS2) sublines and control cell lines (TSGH-C1 and -C2; AGS-C1 and -C2) were established. (A, B) Expression of GLO1 was determined using western blot analysis. β-actin was used as an internal control. Cell migration (C, E) and invasion (D, F) abilities were assayed as described in “Materials and Methods”. Data were presented as folds from at least three independent experiments performed in duplicated. The fold changes (C–F), and differences examined using Mann-Whitney U method to compare values with vector control. ** *P*<0.05.

Our results confirmed high expression of GLO1 in advanced gastric cancer, compared to noncancerous gastric mucosa. To determine whether GLO1 expression is associated with invasiveness of gastric cancer cell lines, the effects of GLO1 depletion using short hairpin (sh)RNA plasmids on tumor cell invasion activities of TSGH or AGS cells were assessed. ShRNA expression vectors encoding the antisense GLO1 sequence were transfected into TSGH and AGS cell lines expressing high levels of endogenous GLO1. GLO1 expression was significantly repressed in TSGH-KG1, -KG2 (0.32- and 0.14-fold) and AGS-KS1, -KS2 (0.26- and 0.21-fold) sublines, respectively, compared with that in cells transfected with the control vectors (C1, C2; Figs. 4A and B). KG1 and KG2 GLO1-depleted cells exhibited significantly (*P*<0.05) reduced migration rates (0.16- or 0.044-fold, respectively) and invasion abilities (0.43- or 0.29-fold, respectively) than control vector-transfected cells (Figs. 4C and D). Similar results were obtained with AGS-KS cells (KS1 and KS2) (Figs. 4E and F). Our results collectively suggest that GLO1 positively regulates the migration and invasion abilities of gastric cancer cells.

### Down-expression of GLO1 results in reduced expression of genes involved in metastasis-associated pathways

To ascertain whether the GLO1 protein affects metastasis-related genes, we compared the genome-wide expression of KG2 and C1. Several genes upregulated (≥1.5-fold) in C1, compared to KG2, were selected. MetaCore™ analysis [28] revealed that the top-ranking molecular pathways altered in KG clones were adhesion_cytokines and adhesion pathways. Proteins [such as matrix metalloproteinase (MMP), CXCL8, and CXCL1] involved in those pathways were down-regulated upon GLO1 silencing. Among the cytokine related pathways, high expression of VEGF, CXCL8, CXCR2, and CXCL1 are associated with cancer metastasis and progression [8], [29]. Previously, Daniel J. *et al.* [30] reported that over-expression of GLO1 could enhance stromal cell-derived factor-1 (SDF-1), CXCR4, and VEGF expression in hypoxic endothelial progenitor cells culture in high glucose. Therefore, we also analyzed the expression of VEGF in KG stable lines.

**Figure 5:**
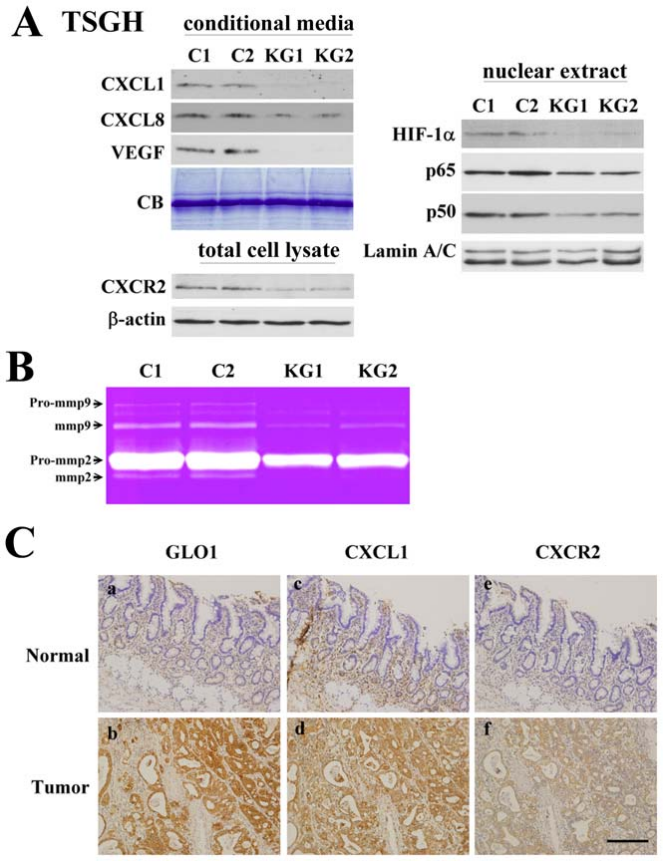
Downstream target genes of GLO1 and their clinical correlations. (A) HIF-1α, NF-kB, VEGF, CXCL8, CXCL1, and CXCR2 protein levels in TSGH cells transfected with GLO1 shRNA (KG1 and KG2) and control shRNA (C1 and C2). The gel was stained with Coomassie blue (CB), which was used as a loading control of conditional media. β-actin was used as an internal control for total cell lysates, and lamin A/C for nuclear proteins. (B) Knockdown of GLO1 suppressed activation of MMP2 and MMP9. Conditional media from C1, C2, KG1 and KG2 cells were collected and subjected to gelatin zymography. (C) Sections of formalin-fixed and paraffin-embedded tissues from three human gastric tumor tissues were immunostained with anti-GLO1 (a and b), anti-CXCL1 (c and d) or anti-CXCR2 antibodies (e and f). Coexpression of GLO1, CXCL1, and CXCR2 proteins detected in human gastric cancer tissues (b, d, and f). Noncancerous gastric mucosa with negative or lower expression of GLO1, CXCL1 and CXCR2 proteins (a, c, and e). Scale bar represents 200 µm.

We further validated the expression patterns of proteins in VEGF or cytokine-related pathways via western blot analysis. The levels of target genes, including CXCL1, CXCL8, CXCR2, and VEGF, were significantly inhibited in TSGH-KG stable cell lines (KG1 and KG2), compared with vector-transfected controls (Fig. 5A). Furthermore, the levels of NF-κB and HIF1-α, well-known transcription factors of pro-angiogenic growth factors (such as CXCL8, CXCL1, and VEGF) [31], were reduced in the nuclei of TSGH-KG stable cell lines, compared with control cells (Fig. 5A). MMP2 and MMP9, the key enzymes for degrading type IV collagen, are believed to play a critical role in tumor invasion and metastasis [32]. Notably, depletion of GLO1 led to marked suppression of MMP2 and MMP9 activities (Fig. 5B). Our data indicate that GLO1 regulates the activation of metastasis-associated signaling pathways in gastric cancer cells. Based on these findings, we propose that GLO1 mediates gastric cancer cell migration and invasion at least partially mediated through activation of CXCL1, CXCL8, and VEGF.

### IHC shows coexpression of GLO1 with CXCL1 and CXCR2 proteins and its over-expression in gastric cancerous tissues

Earlier studies have reported that cytokine receptor interaction and VEGF signaling pathways are associated with malignancy features in gastric cancer [8], [33]. Previously, our group showed a significant association of CXCR2 and CXCL1 over-expression (n = 116) with gastric cancer progression [8]. Consistently, in IHC analysis, a positive significant correlation was found exclusively between the scores of GLO1 and CXCL1 or CXCR2 in cancer tissues (Spearman's correlation coefficient = 0.238 and 0.293; *P* = 0.013 and *P* = 0.003, respectively). Furthermore, immunostaining of consecutive sections revealed significant expression of GLO1, CXCL1, and CXCR2 proteins in tumor epithelial cells [Fig. 5C (b), (d), and (f)], in contrast to no or low expression in noncancerous tissues [Fig. 5C (a), (c), and (e)].

## Discussion

As the second most frequent cause of cancer-related death, gastric cancer remains a challenging disease. Data from the present study demonstrated that upregulation of GLO1 in gastric cancer tissues is significantly associated with tumor progression and advanced stages of the disease. Concordantly, patients with lower GLO1 levels had better disease prognosis. Moreover, qRT–PCR and IHC experiments disclosed a correlation of increased GLO1 expression with local tumor progression and lymph node invasion. We observed a marked decrease in the metastasis and invasion abilities of GLO1-deficient cells, concomitant with reduced levels of several metastasis-associated factors, including VEGF, CXCL1, CXCL8, MMPs, and CXCR2. In the IHC study, a positive significant correlation between GLO1 and CXCL1 expression patterns was observed in resected specimens of gastric cancer. Moreover, elevated GLO1 and concomitant CXCL1 over-expression in patients with gastric cancer were significantly correlated with survival. Our results provide direct evidence supporting the involvement of GLO1 in gastric cancer progression and the might through alternation of its downstream migration and invasion pathways.

High expression of GLO1 has been linked to several cancers [16], [17]. Recent studies have suggested a vital role of GLO1 in several cancer types in the removal of methylglyoxal (MG), which is considered carcinostatic, resulting in the development of GLO1 inhibitors as anti-tumor agents [34], [35]. Thus, high expression of GLO1 is involved in cancer cell resistance to apoptosis induced by anti-tumor agents [36]. Sakamoto *et al.* [36] proposed that GLO1 is not only a tumor but also a drug resistance marker. Consistently, the GLO1 knockdown-clones were sensitive to several chemotherapy agents such as camptothecin, etoposide (data not shown). Clinically, our results indicate that GLO1 is highly expressed in gastric cancer and significantly associated with tumor progression and advanced stages of the disease.

Glycolytic alterations in cancer cells represent a metabolic adaptation to hypoxic tumors via the action of hypoxia-induced transcription factor (HIF-1) [37], [38]. In addition, the transcription factors, HIF1-α and NF-κB, play crucial roles in various processes, such as inflammation, microbial killing and cancer progression [39]. Tumor hypoxia appears to be strongly associated with tumor propagation, malignant progression, and resistance to therapy. The HIF-1α pathway is clearly involved in carcinogenesis of gastric cancer [39]. In addition, immunohistochemical expression of HIF-1α target genes (Glut1, VEGF, CA9, iNOS) is associated with gastric tumor progression [40]. Among the oncogenic pathways, NF-κB signaling is elevated in a significant proportion of gastric cancers [9]. Moreover, several lines of evidence support crosstalk between the NF-κB and HIF-1α signaling pathways [41]. In our experiments, levels of both HIF-1α and NF-κB were reduced in nuclei upon GLO1 silencing in gastric cancer cell lines, along with downstream target genes (VEGF, CXCL1, CXCL8, MMPs).

In our study, abnormally elevated GLO1 expression was associated with progressive phenotypes, such as gastric wall invasion, lymph node metastasis, pathological stage, and lymphatic invasion. Previously, we reported that elevated CXCL1 and CXCR2 in gastric cancer is associated with tumor progression, and that the plasma CXCL1 level may be a useful circulating biomarker for gastric cancer diagnosis [8]. Here, we have obtained direct clinical evidence of a strong correlation between the expression patterns of GLO1 and CXCL1 or CXCR2. GLO1 expression may be associated with activation of cytokine receptor associated and VEGF signaling pathways, representing a potential mechanism for enhanced motility and invasive ability. Our results confirm over-expression of GLO1 in gastric carcinoma and its strong association with advanced stages and poor prognosis. Earlier studies by our group have shown that SPARC [18], CLIC1 [42], SLPI [43], CXCL1, CXCL8, and CXCR2 are highly expressed and associated with advanced stages and poor prognosis of gastric cancer [8]. These novel potential biomarkers may therefore be applied to improve the specificity and sensitivity of gastric cancer diagnosis. Further development and confirmation of the utility of these markers in larger patient cohorts will potentially lead to clinical applications.

## Supporting Information

Figure S1 The specificity of in-house GLO1 was validated by western blot analysis. Rabbit polyclonal anti-GLO1 antibody (right image) and negative control pre-immune sera (left image) were used. The GLO1 protein level was determined in TSGH-C1 and -KG2 gastric cell lines with the in-house GLO1 antibody. β-actin was used as an internal control for total cell lysates.
(TIF)
