# Correction: Glyoxalase-I Is a Novel Prognosis Factor Associated with Gastric Cancer Progression

**DOI:** 10.1371/annotation/5e2c310d-9811-4d81-a8d5-2701953f2f46

**Published:** 2012-09-12

**Authors:** Wan-Li Cheng, Ming-Ming Tsai, Chung-Ying Tsai, Ya-Hui Huang, Cheng-Yi Chen, Hsiang-Cheng Chi, Yi-Hsin Tseng, Im-Wai Chao, Wei-Chi Lin, Sheng-Ming Wu, Ying Liang, Chia-Jung Liao, Yang-Hsiang Lin, I-Hsiao Chung, Wei-Jan Chen, Paul Y. Lin, Chia-Siu Wang, Kwang-Huei Lin

A second corresponding author, Chia-Siu Wang, was not signified. The author Chia-Siu Wang can be contacted at wangcs@adm.cgmh.org.tw

Also, there is an error in Figure 3. The correct Figure 3 can be seen here: 

**Figure:**
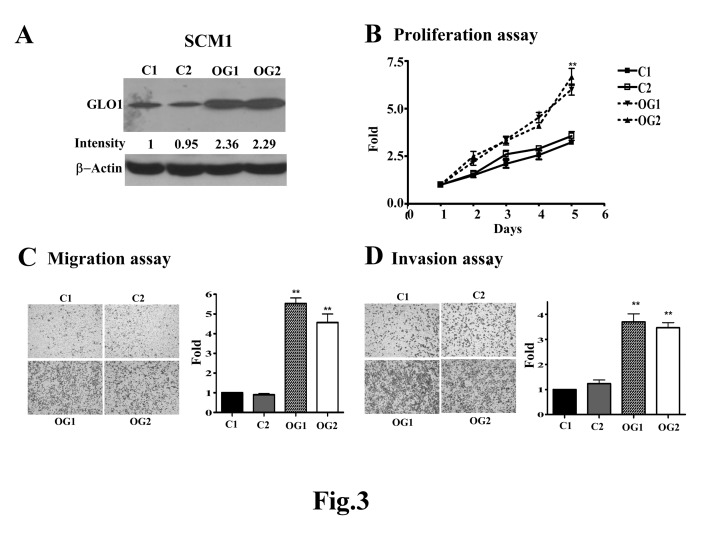


[^] 

