# Supplementary figures and images for: Glyoxalase-I Is a Novel Prognosis Factor Associated with Gastric Cancer Progression
Source: PLoS One. 2012 Mar 29;7(3):e34352. doi: 10.1371/journal.pone.0034352 (PMC3315534; doi:10.1371/journal.pone.0034352)

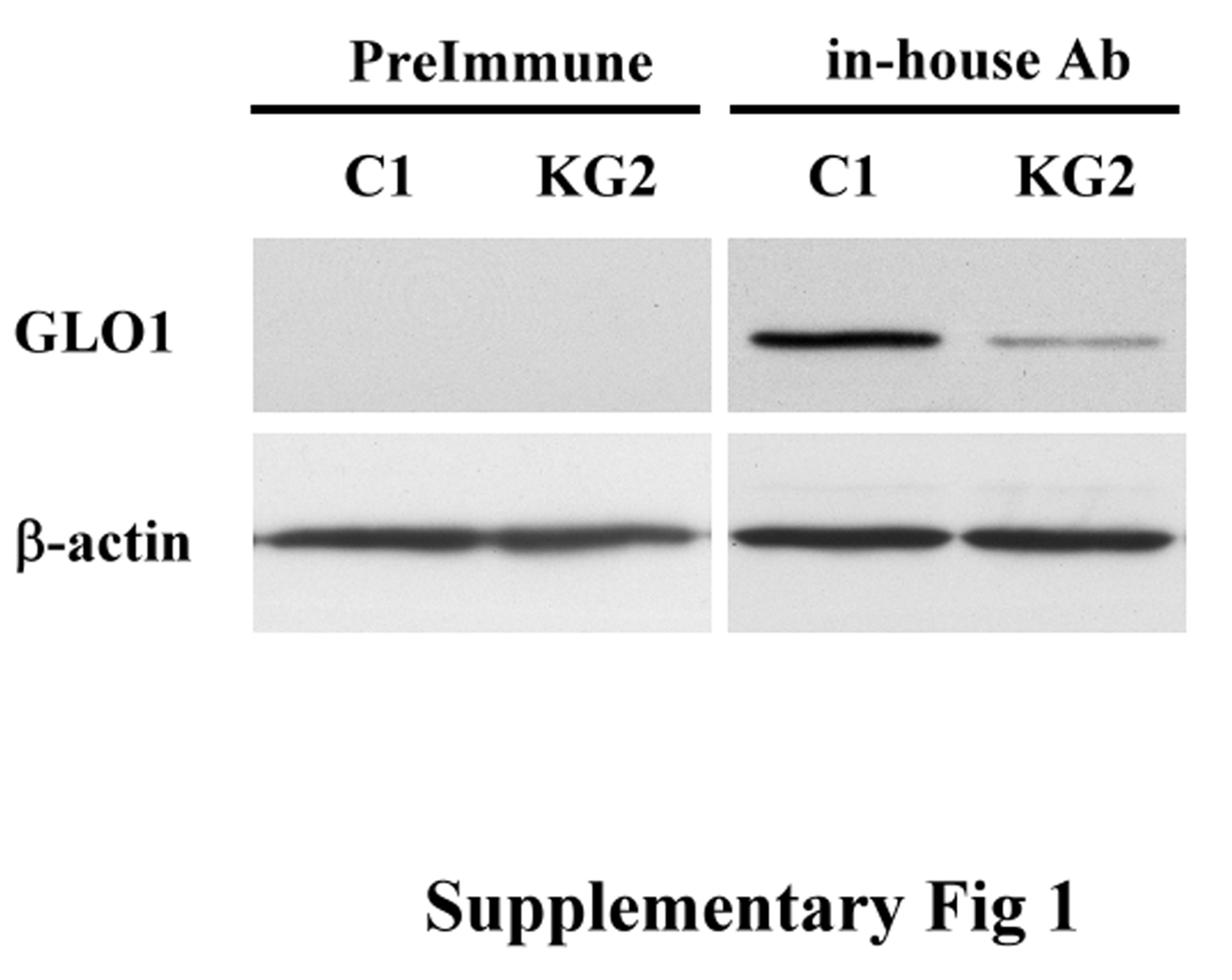

Supplement: Figure S1 — The specificity of in-house GLO1 was validated by western blot analysis. Rabbit polyclonal anti-GLO1 antibody (right image) and negative control pre-immune sera (left image) were used. The GLO1 protein level was determined in TSGH-C1 and -KG2 gastric cell lines with the in-house GLO1 antibody. β-actin was used as an internal control for total cell lysates. (TIF) [file pone.0034352.s001.tif]
